# Vesicular stomatitis virus G protein transmembrane region is crucial for the hemi-fusion to full fusion transition

**DOI:** 10.1038/s41598-018-28868-y

**Published:** 2018-07-13

**Authors:** Yali Ci, Yang Yang, Caimin Xu, Lei Shi

**Affiliations:** 1 State Key Laboratory of Medical Molecular Biology, Institute of Basic Medical Sciences, Chinese Academy of Medical Sciences & Peking Union Medical College, School of Basic Medicine, Beijing, China; 2 Department of Biochemistry and Molecular Biology, Institute of Basic Medical Sciences, Chinese Academy of Medical Sciences & Peking Union Medical College, School of Basic Medicine, Beijing, China

## Abstract

Viral fusion proteins are essential for enveloped virus infection. These proteins mediate fusion between the virus envelope and host cellular membrane to release the viral genome into cells. Vesicular stomatitis virus G (VSV G) protein is a typical type III viral fusion protein. To study the mechanism of VSV G protein mediated membrane fusion, we set up a cell-cell fusion system in which cells are marked by different fluorescent proteins. Taking advantage of this system, we performed real-time recording and quantitative analysis of the cell fusion mediated by VSV G. We found that the time scale required for VSV G mediated cell-cell fusion was approximately 1–2 minutes. Next, we specifically examined the function of the transmembrane (TM) region of VSV G protein in membrane fusion by replacing the TM region with those of other fusion proteins. The TM region replacements dramatically impaired VSV G protein function in the cell-cell fusion assay and diminished VSV G mediated lentivirus and recombinant VSV infection efficiency. Further experiments implied that the TM region played a role in the transition from hemi-fusion to full fusion. Several residues within the TM region were identified as important for membrane fusion. Overall, our findings unraveled the important function of the TM region in VSV G mediated viral fusion.

## Introduction

Membrane fusion is a universal and important biological phenomenon involved in multiple physiological and pathological processes, ranging from cell fusion and organelle dynamics to vesicle trafficking and viral infection^1–5^. Without exception, all of these fusion events are driven by membrane fusion proteins, also known as fusogens^6^. The common fusion process mediated by fusion proteins consists of a series of steps that includes the approach of two opposing lipid membranes, breaking the lipid bilayers, and finally merging the two lipid bilayers into one^7^. Much of our understanding of membrane fusion comes from studies of vesicle fusion, which is driven by a special kind of protein called SNARE^8^. The SNARE proteins on vesicles (v-SNARE) and those on target membranes (t-SNARE) provide not only recognition specificity but also the energy needed for vesicle fusion^9^.

Viral fusion is another important fusion event. Enveloped viruses that are encapsulated by membranes derived from host cells release genomes after the fusion between viral envelope and host cellular membrane^10^. Viral fusion proteins dominate the uncoating stage^11^. According to their structural characteristics, viral fusion proteins are classified into three types: I, II and III^11^. Despite longstanding knowledge of viral fusion proteins, the underlying fusion mechanism remains mysterious. One such previously identified type III viral fusion protein is vesicular stomatitis virus G protein (VSV G)^12^. Previous studies have revealed that VSV G triggered membrane fusion in acidic environments relies on reversible conformational changes, which return to their original state under neutral conditions^13^. VSV G structures under neutral and acidic conditions, corresponding to pre- and post-fusion stages respectively, have been resolved^14,15^. However, there are still some unanswered questions concerning how the VSV G protein drives membrane fusion, the special roles of individual domains and how these domains cooperate with each other.

Different membrane fusion proteins function in different ways but also share some common rules. A few domains and motifs have already been proven to be crucial for the fusion process, including the coiled-coil domain/SNARE motif of SNARE proteins, the fusion peptide or loop in viral fusion proteins and so on^16–18^. Additionally, the transmembrane (TM) region, which is the fusogen anchor on the membrane, may also participate in fusion. It has been reported that the TM region is the mechanical element that exerts force on the lipid membrane. Solid evidence supports the notion that the TM regions of SNARE proteins participate in fusion pore formation and stability^19–21^.

Previous studies demonstrated that TM replacement by other sequences did not affect VSV G protein fusion ability^22^. However, evidence from some other viral fusion proteins and SNAREs indicates that the TM regions are essential for membrane fusion^23–25^. To interpret these apparently conflicting results, we studied the function of the VSV G protein TM region through cell-cell fusion assays and viral infection assays. We report here that the TM region was important for VSV G protein mediated membrane fusion and viral infection. Replacement of the TM region impaired the fusion function of VSV G and blocked the fusion process at the hemi-fusion stage. Moreover, we identified several fusion-related residues in the TM region, implying that the role of TM in membrane fusion is sequence dependent. Our findings provide new insight into the mechanism of VSV G mediated virus fusion and suggest a common rule in which the TM region acts as a key element for the fusion activity of versatile membrane fusion proteins.

## Results

### VSV G mediated cell-cell fusion was complete within minutes

**Figure 1:**
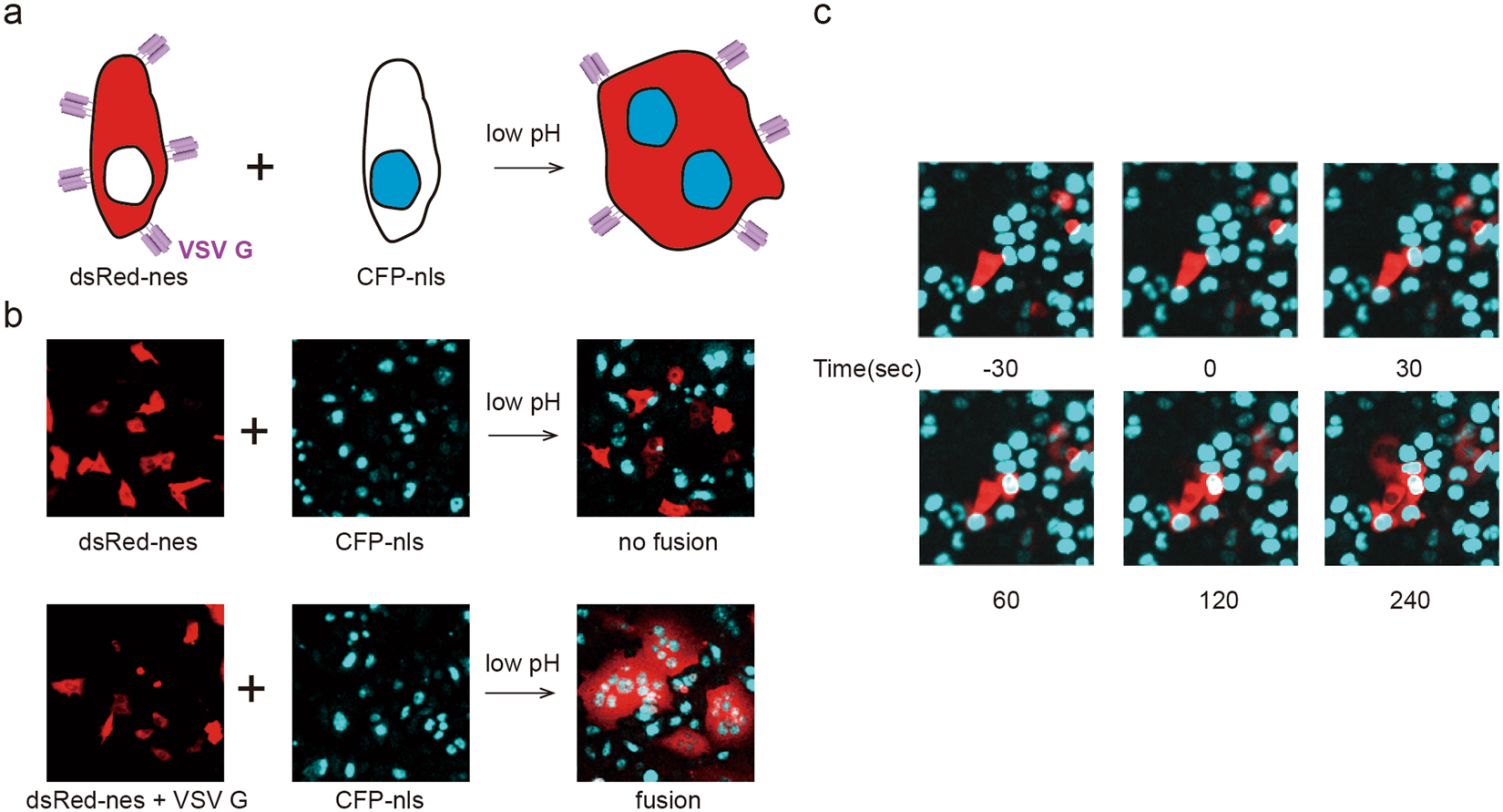
Cell-cell fusion mediated by the VSV G protein. (**a**) Schematics show the cell-cell fusion assay: cells expressing VSV G proteins and cytosol-localized dsRed fused with cells expressing nucleus-localized CFP upon low pH stimulation. The fusion cells contain multiple cyan nuclei surrounded by red cytosol. (**b**) VSV G protein mediated HeLa cell-cell fusion. No fusion was observed in the control experiment in the absence of VSV G proteins after low pH treatment (upper panel), while many fused cells were observed in the VSV G expression group under the same conditions (bottom panel). (**c**) The time course of VSV G protein mediated cell-cell fusion. VSV G proteins induced cell-cell fusion, which occurred quickly after treatment with acidic buffer.

It is well known that VSV G proteins induce cell-cell fusion upon stimulation by low pH. Syncytium formation assays have been used to monitor the fusion activity of VSV G, but their quantification inaccuracy and inability to achieve dynamic monitoring limit their applications. Here, we set up a cell-cell fusion assay to uncover the details of the VSV G induced fusion process^26^. HeLa cells were co-transfected with plasmids encoding VSV G and dsRed with a nuclear export signal (dsRed-nes). As a result, VSV G proteins were expressed on the cell surface, and the cytosol was labeled by dsRed (Fig. 1a). Another group of HeLa cells was labeled by CFP with a nuclear localization signal (CFP-nls) (Fig. 1a), resulting in blue nuclei. After overnight co-culture, these two groups of HeLa cells were treated with acidic buffer (pH 6.0) for a short duration (1 min), and some adjacent cells fused with each other. As shown in Fig. 1a,b, the fused cells had multiple blue nuclei surrounded by red cytosol. Note that many giant cells with more than 4 nuclei manifested multiple rounds of fusion occurs (Fig. 1b). Few fusion cells were observed in the control group without VSV G expression (Fig. 1b). Real-time recording is an additional advantage of this cell-cell fusion assay. We found that cell-cell fusion mediated by VSV G proteins occurred very quickly. After treatment with acidic buffer, red cytosol (dsRed) diffused from one cell to another within 1 minute (Fig. 1c and Movie S1). This time course was similar to that of VSV-lipid bilayer fusion, which showed that the mean time required for VSV G induced hemi-fusion is approximately 25–30 seconds^27^. Given the time delay of dsRed protein diffusion from one cell to another through a narrow fusion pore in our system, the actual time required for membrane fusion should be even shorter, probably by a few seconds. With these quantitative and kinetic results, we demonstrated that the VSV G protein triggered membrane fusion in a quick and effective manner.

### Replacement of the TM region had no effect on VSV G protein expression levels, plasma membrane localization and extracellular conformation

**Figure 2:**
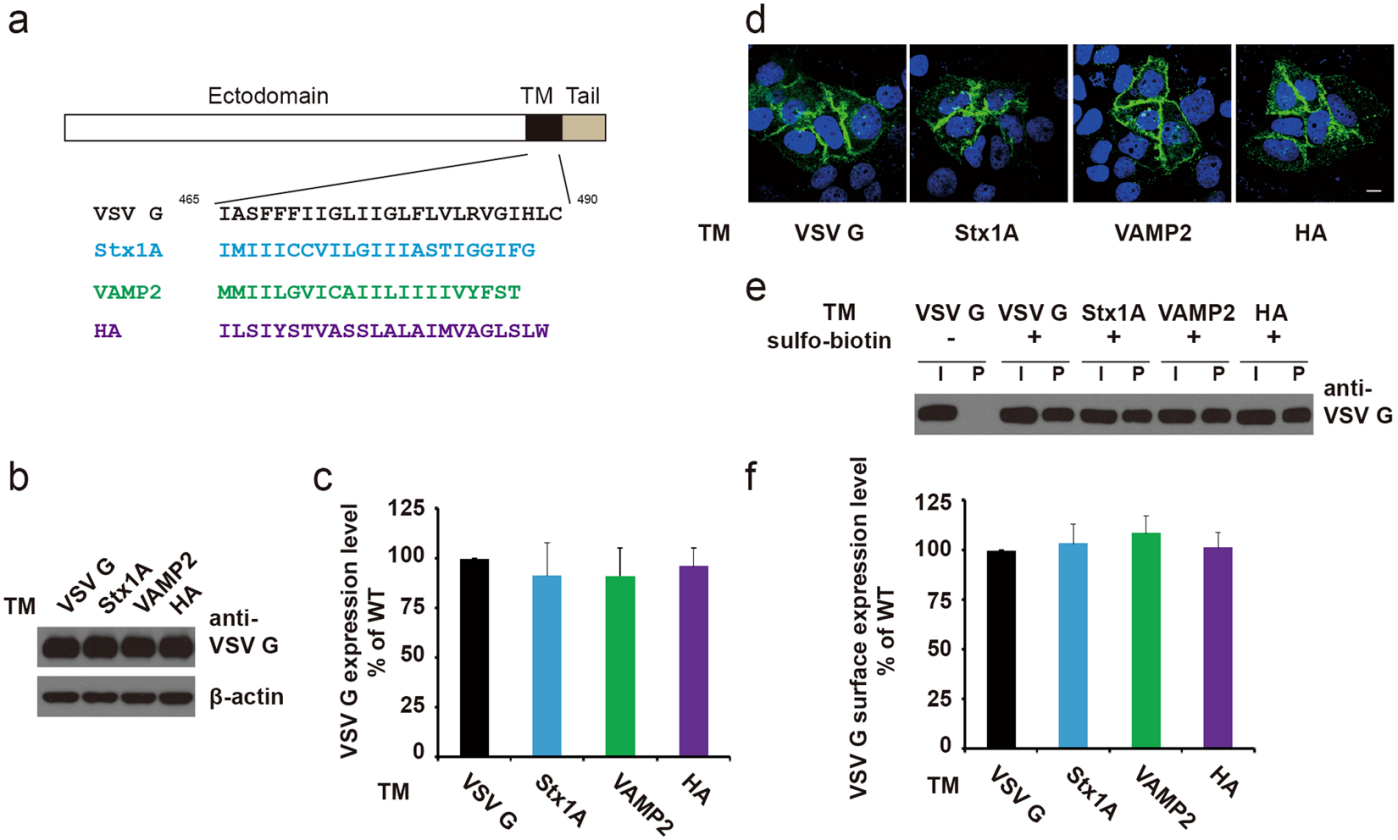
Replacement of the VSV G TM region with TM regions from other membrane fusion proteins resulted in similar protein expression levels and plasma membrane localization. (**a**) Sequences of the TM regions of VSV G, Syntaxin 1 A, VAMP2 and influenza HA. (**b** and **c**) VSV G TM replacement did not lead to dramatic changes in the expression levels of VSV G in HeLa cells. (**d**) WT and chimeric VSV G localized onto the plasma membrane. HeLa cells were transfected with plasmids encoding WT or chimeric VSV G proteins fused with EGFP. (**e** and **f**) Biotinylation assays to quantify membrane-localized WT or chimeric VSV G proteins. Total VSV G levels in cells (I: input) and membrane-localized G protein levels (P: pull down by streptavidin resin) were determined by western blot. The ratio of biotinylated VSV G to total G protein was calculated. Compared to WT VSV G, chimeric mutants showed similar levels of membrane localization. Mean ± SEM.

Several pioneering studies on other fusion proteins reported a principal role for the TM domain in the fusion process^21,23,24^. Although the data from a VSV G study showed that TM replacement did not affect fusion activity^22^, the lack of a coherent understanding of TM function in membrane fusion prompted us to explore whether TM regions from different fusion proteins are equivalent to one another. We replaced the TM domain of the VSV G protein with those from other fusion proteins: two from neuronal SNARE proteins (Syntaxin1A, accession no. NM_053788, and VAMP2, accession no. BC055105) and one from a type I viral fusion protein (influenza HA, accession no. AAW72226). These TM regions have been previously proved to be necessary for membrane fusion^21,24^. The TM regions have similar lengths, but their sequences are non-conserved, as shown in Fig. 2a. We first examined whether these replacements affected VSV G protein expression levels in the cell. Chimeric VSV G proteins had expression levels that were similar to that of the wild type (WT) (Fig. 2b,c). Due to the requirement of VSV G plasma membrane localization during cell-cell fusion, we next examined the subcellular localization of these chimeric VSV G proteins. Fluorescent imaging showed that TM replacement did not influence the plasma membrane localization of VSV G (Fig. 2d). To further quantify the transport efficiency of VSV G on the plasma membrane, we performed surface protein biotinylation assays to analyze the ratio of plasma membrane localized G proteins to total G proteins, as previously described^28^. Our results revealed that WT and chimeric VSV G had similar ratios of surface to total VSV G, suggesting that TM replacement did not affect VSV G transport onto the plasma membrane (Fig. 2e,f). Similar signals in a cell surface ELISA assay using a VSV G antibody showed that TM replacement did not affect the conformation of VSV G ectodomain (Table. S1). In summary, these results demonstrated that TM replacement did not affect VSV G protein expression levels, plasma membrane localization and ectodomain conformation.

### Replacement of the VSV G protein TM region decreased cell-cell fusion efficiency

**Figure 3:**
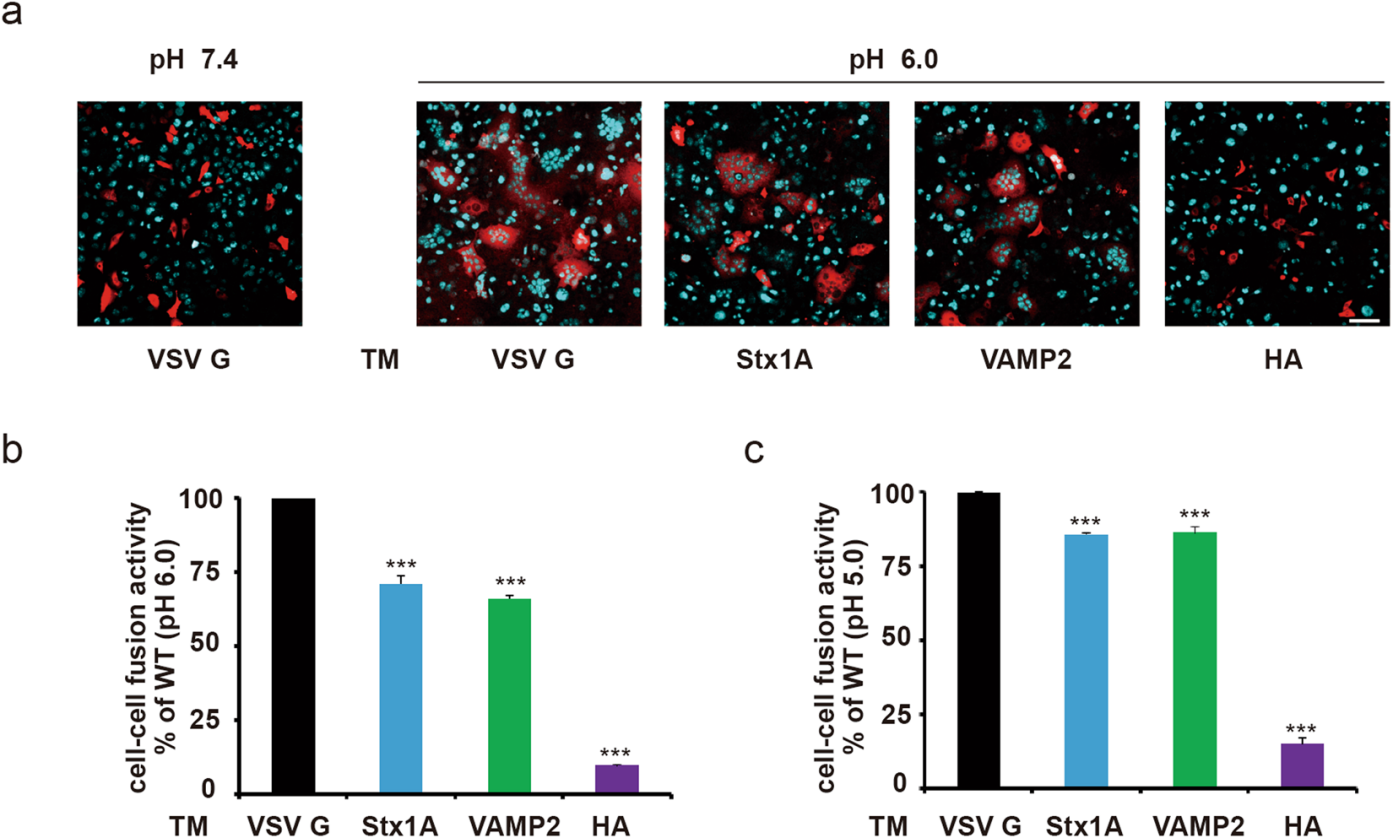
TM replacement dramatically reduced VSV G mediated cell-cell fusion. (**a**) Cell-cell fusion mediated by WT or chimeric VSV G proteins. Scale bar, 80 μm. (**b**) TM replacement dramatically reduced VSV G mediated cell-cell fusion efficiency (P < 0.001). Cell-cell fusion efficiency is defined as the ratio of fused cells to total dsRed-labeled cells. (**c**) Lower pH (5.0) conditions did not rescue the fusion deficiency of the chimeric mutants. When buffer with a lower pH (5.0) was used as a stimulus, fusion efficiency increased in all groups. However, TM replacement still led to significant decreases in cell fusion efficiency (P < 0.001).

Based on the similar VSV G expression levels and cell surface localization observed between WT and chimeric VSV G, we compared their fusion abilities. A cell-cell fusion assay was performed with HeLa cells expressing WT or chimeric VSV G proteins. All three chimeric VSV G proteins induced remarkable defects in cell fusion. Replacement with TM regions from two SNARE proteins resulted in a reduction of approximately 30%, while replacement with the HA TM region nearly abolished the fusion activity of VSV G (Fig. 3a,b). These results indicated that the VSV G TM region played an important role in membrane fusion, and its function depended on its sequence. Next, we further decreased the working pH to 5.0 to mimic physiological pH conditions in the late endosome and lysosome, and the fusion efficiency increased in all groups, but the fusion efficiencies of the chimeric mutants were still lower than that of the WT (Fig. 3c). Thus, the fusion defect caused by TM replacement could not be rescued under lower pH conditions, although VSV G conformational changes are pH-dependent. Taken together, our findings indicated the importance of the TM region in the fusion activity of VSV G and highlighted the lack of equivalent TMs from different fusion proteins.

### TM replacement blocked fusion at the hemi-fusion stage

**Figure 4:**
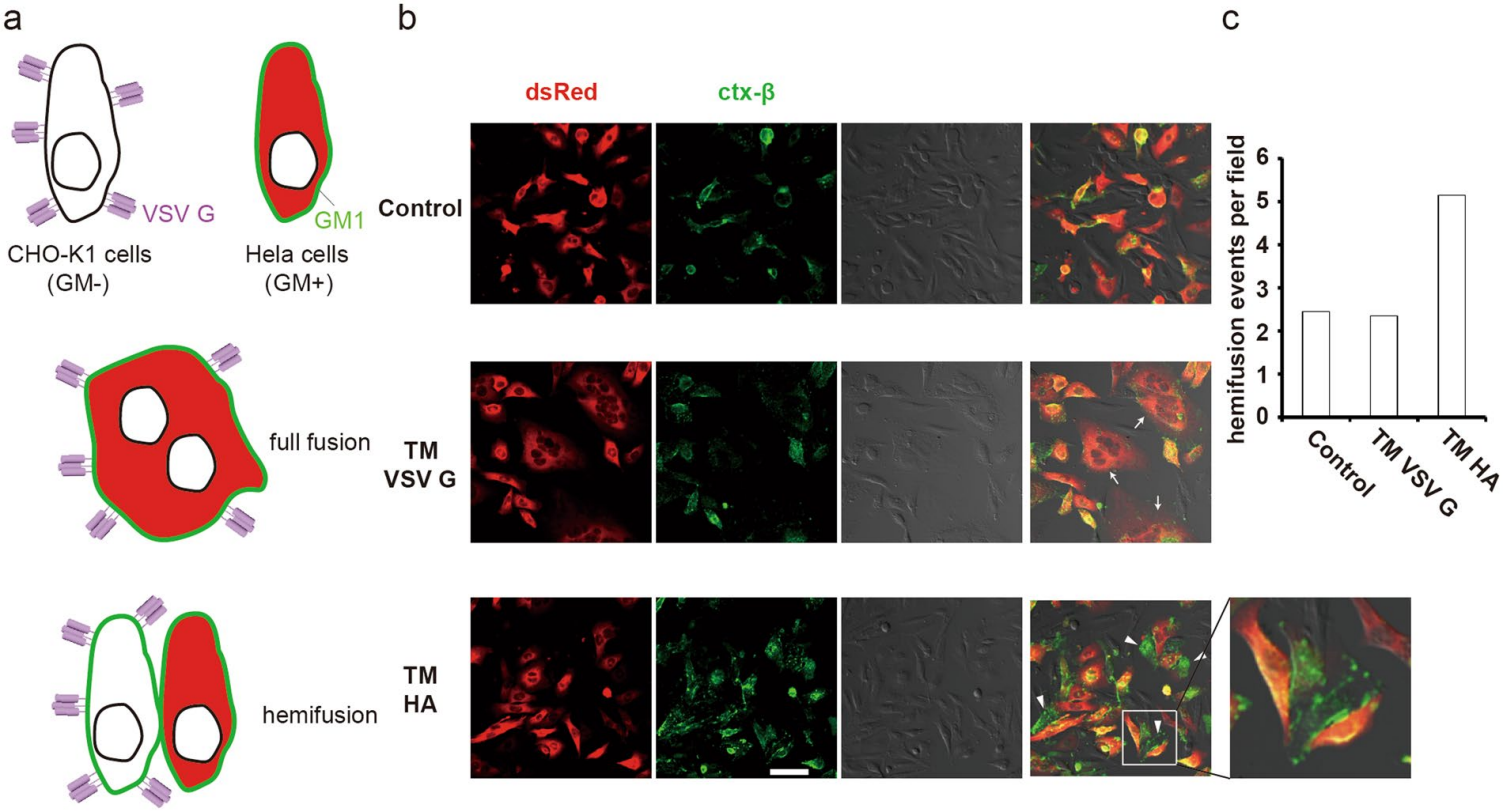
TM replacement blocked fusion at the hemi-fusion stage. (**a**) Schematics show a cell-cell fusion assay model for detecting hemi-fusion events. CHO-K1 cells (GM1 negative) expressing VSV G were co-cultured with HeLa cells (GM1 positive) expressing dsRed-nes. FITC-cholera toxin β-subunit (green) was used to label GM1 on the cell membrane. Fused cells with multiple nuclei show a red cytosol surrounded by a green membrane. Hemi-fused CHO cells present only green plasma membrane without red cytosol, and adjacent HeLa cells have red cytosol and green membrane. (**b**) Chimeric VSV G with HA TM blocked cell fusion at the hemi-fusion stage. CHO-K1 cells expressing WT or chimeric VSV G fused with HeLa cells stably expressing dsRed-nes. Arrows indicate full-fused cells (middle panel), and arrowheads indicate hemi-fused cells (lower panel). Scale bar, 50 μm. (**c**) Quantification of hemi-fusion events. Hemi-fusion events in each field were counted (20 random fields), and the average number is shown.

We already know that TM replacement disrupts VSV G mediated cell-cell fusion, but the details of this fusion process remain unresolved. Overall, the physical process of membrane fusion is similar for different fusogens, consisting of the approach of opposing membranes, the intermediate hemi-fusion stage and final membrane merging. Hemi-fusion is an important intermediate state characterized by the merging of the outer leaflets of opposing membranes in the absence of lipid mixing from the inner leaflets, opening of the fusion pore and content exchange^29^. We set up another cell fusion assay to capture hemi-fusion events^30^. A previous study showed that HeLa cells possess ganglioside (GM1) on the outer leaflets of the plasma membrane, whereas CHO-K1 cells do not^31^. We expressed cytosolic dsRed in HeLa cells and VSV G in CHO-K1 cells and then induced the fusion of these two groups of cells under low pH condition. With hemi-fusion between the CHO-K1 and HeLa cells, the lipid mixing of outer leaflets caused GM1 transfer from HeLa cells to CHO-K1 cells, while cytosolic dsRed diffusion did not occur due to the lack of fusion pore opening. Therefore, the CHO-K1 cells that were halted at the hemi-fusion stage had GM1 on the membrane (marked by FITC-labeled cholera toxin β-subunit) but no red color in the cytosol (Fig. 4a). On the contrary, full-fusion events result in multi-round fused cells with more than one nucleus labeled by both red cytosol and green plasma membrane (Fig. 4a). As shown in Fig. 4b,c, VSV G-HA TM-expressing CHO-K1 cells were characterized by a green membrane but not by a red cytosol (bottom panel), suggesting the occurrence of hemi-fusion, although at a low frequency. WT VSV G mostly induced full-fusion events between CHO-K1 and HeLa cells, characterized by both a red cytosol and a green membrane (Fig. 4b,c, middle panel). Thus, the cell fusion deficiency exhibited by chimeric VSV G with HA TM is attributable to arrest at the hemi-fusion stage. Hemi-fusion blockage by VSV G-HA TM indicated that fusion initiation was not affected; however, TM is indispensable for the transition from hemi-fusion to full fusion.

### VSV G protein TM replacement impaired lentivirus and VSV infection

**Figure 5:**
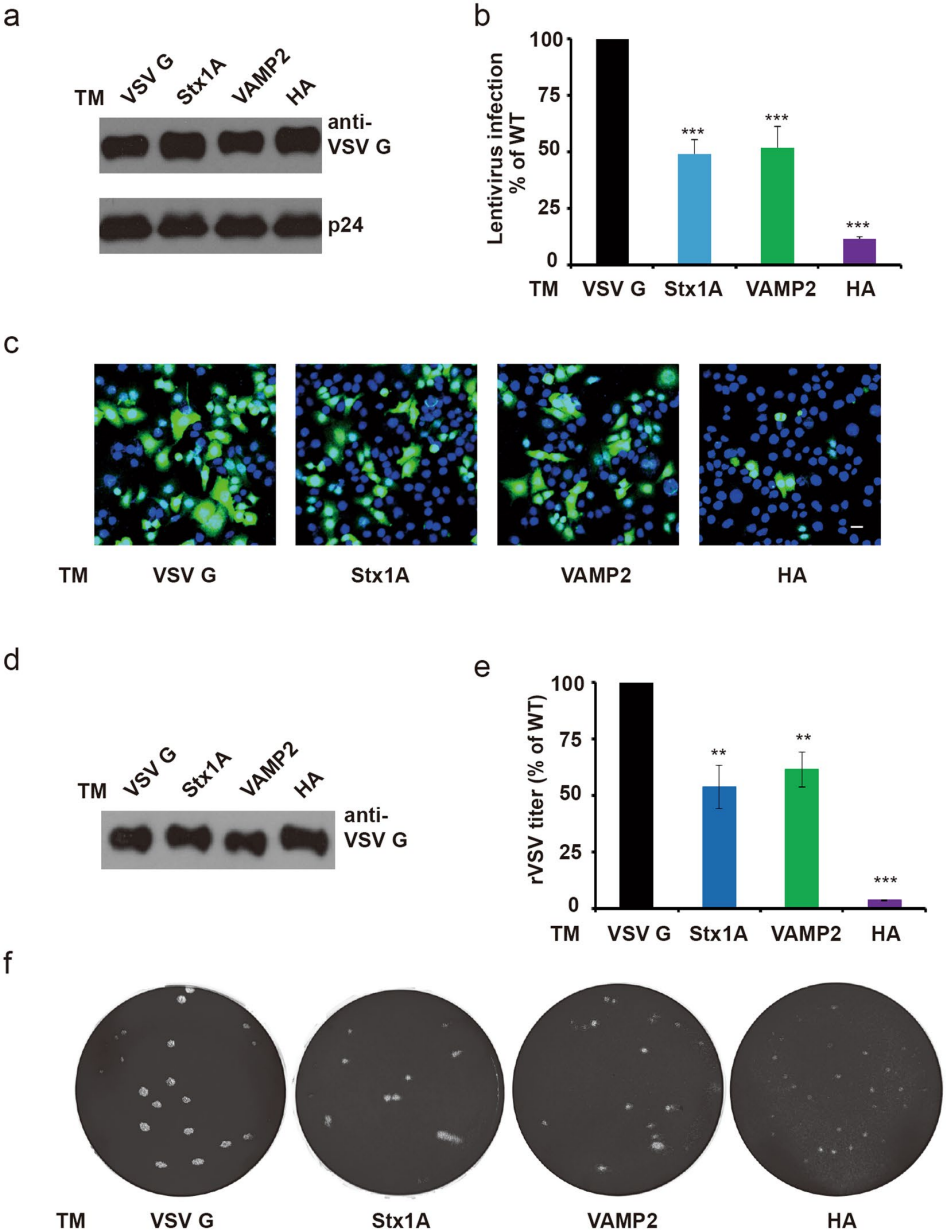
TM replacement impaired VSV G protein mediated virus infection. (**a**) WT or chimeric VSV G proteins were packed into lentiviruses at similar levels. HEK293 T cells were co-transfected with pLVTHM, pMD 2.G (WT or chimeric VSV G) and psPAX2 to generate lentivirus. Lentivirus was collected and analyzed by western blot with a VSV G antibody. The capsid protein P24 was used as a normalization control. (**b** and **c**) TM replacement remarkably attenuated VSV G induced lentivirus infection. HeLa cells were infected by lentivirus enveloped with WT or chimeric VSV G. A GFP gene was used as a reporter of virus infection. Lentivirus-infected cells (GFP positive) were analyzed by flow cytometry. The cell infection efficiency decreased dramatically when VSV G TM was replaced by TM from Stx1A, VAMP2 or HA (***P < 0.001). Scale bar, 30 μm. (**d**–**f**) TM replacement of VSV G reduced the infectivity of rVSV. (**d**) 293 T cells were co-transfected with pBS-N, pBS-P, pBS-L, pCAG-T7 and WT or mutant pVSV to produce rVSV. WT or chimeric VSV G packaging into rVSV was determined by western blot. (**e** and **f**) The infection efficiency of rVSV enveloped with chimeric VSV G was lower than that of WT rVSV. Plaque assays were performed by serial dilution of the indicated virus. The plaque morphologies of rVSV with WT or Syntaxin, VAMP2 TM-VSV G (10^−4^ dilution) and rVSV with HA TM-VSV G (10^−3^ dilution) are shown in (**f**).

Fusion is an essential step in enveloped virus infection. In addition to VSV, VSV G was also constructed into lentivirus for gene delivery because of its wide host tropism^32^. Thus, we detected the infection efficiency of lentiviruses packaged with VSV G mutants. WT or chimeric VSV G protein was packaged into the lentivirus, which was verified by western blot. P24 protein, a capsid protein, was used as an internal control. Similar amounts of WT or chimeric VSV G on viruses indicated that TM replacement did not affect viral packaging (Fig. 5a). Equal amounts of lentivirus carrying WT or chimeric VSV G resulted in different viral infection efficiency (GFP as a reporter). TM replacement in VSV G dramatically reduced lentivirus infection. Quantitative analysis by flow cytometry determined the percentage of lentivirus-infected cells (Figs 5b,c and S2). Compared to lentiviruses carrying WT VSV G, lentiviruses carrying VSV G mutants with SNARE TM replacement induced a reduction in infection efficiency of approximately 50%, and mutants with HA TM replacement induced a reduction of more than 80%. Then, we returned to original infection model and constructed recombinant VSV with the TM replaced VSV G. The recombinant VSV was generated by co-transfection with a pVSV genome plasmid containing WT or mutated VSV G, three helper plasmids (pBS-N, pBS-P, pBS-L) and pCAG-T7 (encoding T7 polymerase) into 293 T cells^33^. The amount of recombinant VSV (rVSV) was normalized by VSV G, and then WT and VSV G mutated rVSV were titered in a plaque assay (Fig. 5d). rVSV containing VSV G TM replacement mutants notably impaired rVSV infectivity (Fig. 5e,f), which is consistent with the cell-cell fusion data described above. Immunostaining showed that cells took up rVSV with WT or mutated VSV G, and similar amounts of viral RNA in WT and mutated rVSV infected cells at 1 h post infection implied the equivalent efficiency of viral entry (Fig. S3). Thus, compromised viral infection was derived from attenuated VSV G fusion function. With these two kinds of virus, we confirmed that the function of the TM region in VSV G mediated fusion is important for viral infection.

### Sequence dependency of VSV G TM function

**Figure 6:**
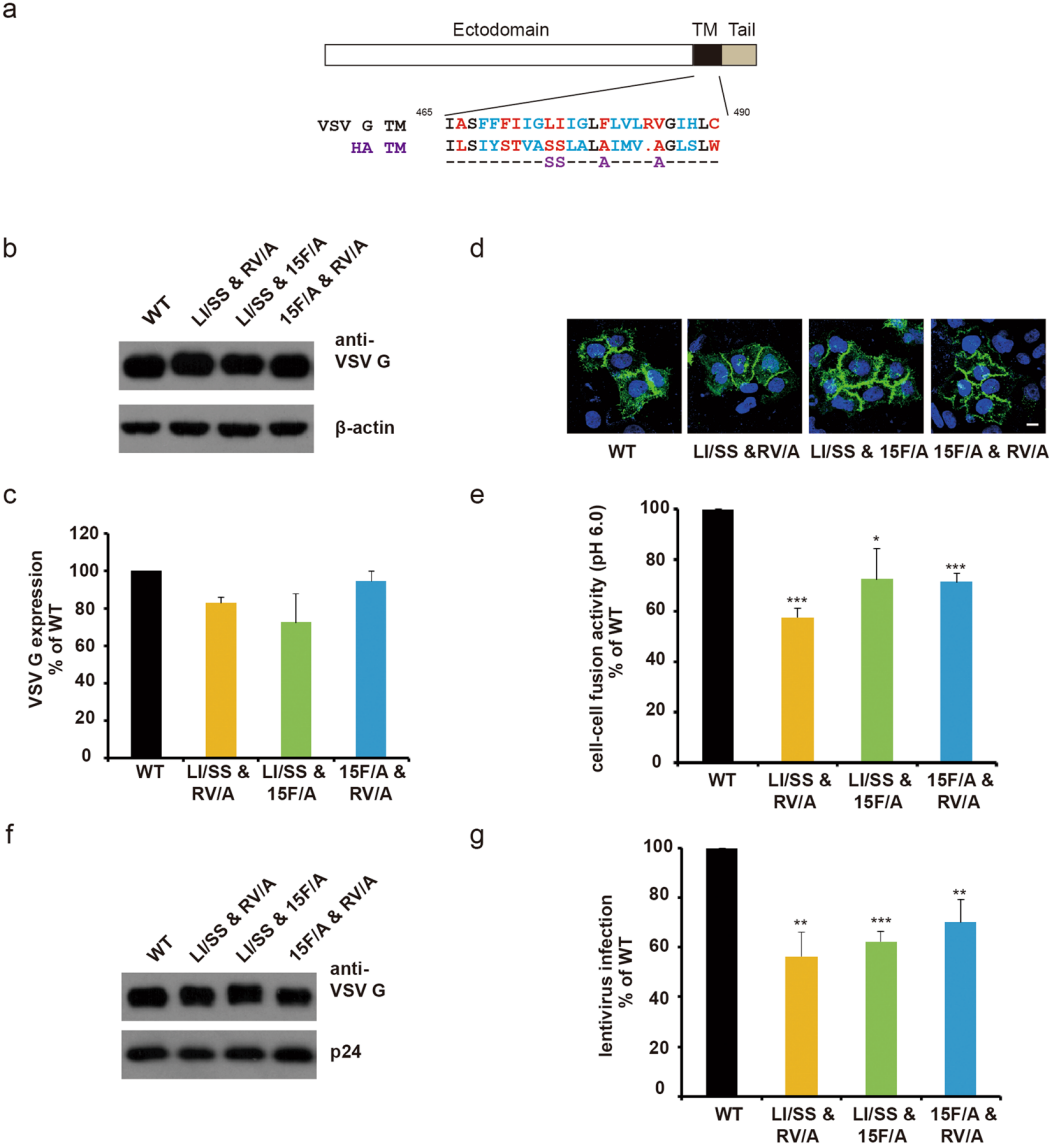
The fusion capacity of the VSV G protein was sequence dependent. (**a**) Amino acid sequence alignment of TMs from VSV G and influenza HA. Black: identical, blue: homologous, red: non-conserved. (**b** and **c**) WT and mutated VSV G had similar expression levels. (**d**) Point mutations in TM did not affect the surface localization of VSV G. The localization of WT and mutated VSV G-EGFP fusion proteins was analyzed by confocal microscopy. Scale bar, 10 μm. (**e**) Point mutations in the TM region impaired the cell-cell fusion activity of VSV G. (**f**) WT and mutated VSV G proteins were packaged into lentiviruses at similar levels. (**g**) Point mutations in the TM region decreased the lentivirus infection efficiency mediated by VSV G. HeLa cells were infected by lentivirus containing WT or mutated VSV G and analyzed by flow cytometry. Asterisks indicate statistically significant differences (*P < 0.05; **P < 0.01; ***P < 0.001).

Our results showed that the TM regions of other fusion proteins did not fulfill the functions of VSV G native TM, suggesting the importance of the TM region. An earlier study showed that glycine in the TM region is critical for the fusion of VSV G and other viral fusion proteins^34^. We next attempted to identify other key residues beyond glycine by mutagenesis analysis. Sequence alignment revealed the homology of the VSV G and HA TM regions (Fig. 6a). We mutated variant residues according to this alignment. Some mutants displayed defects in either expression, membrane localization or lentivirus packaging (Fig. S4). Other single point mutations had normal expression and membrane localization and displayed fusogenic abilities similar to that of WT VSV G in the cell fusion assay. The failure of single point mutation screening prompted us to construct multi-site mutants. Among these mutants, three (LI/SS&RV/A, LI/SS&15 F/A, 15 F/A &RV/A) affected neither the expression nor the localization of the VSV G protein (Fig. 6b–d). However, these mutations led to the attenuation of both cell-cell fusion and viral infection (Fig. 6e–g and S5). These findings demonstrated that L474, I475, F479, R483 and V484 in the TM region are important for VSV G mediated membrane fusion.

## Discussion

Membrane fusion is an essential step for enveloped virus infection. Distinct viral fusion proteins dominate this process through diverse molecular mechanisms. The VSV G protein is one of the earliest identified viral fusion proteins. However, the underlying mechanisms to drive membrane fusion are not fully understood. Here, we set up a cell-cell fusion assay by labeling two groups of cells with different fluorescent proteins to perform quantitative analysis. We recorded the cell fusion process via real-time imaging and reported that VSV G mediated cell fusion occurs in a very short timescale (seconds to minute). This time course is similar to that reported by earlier studies investigating viral fusions with lipid bilayers or cells by single particle imaging^27,35,36^. Compared to the timescale of SNARE mediated vesicle fusion (micro- to milliseconds), our cell fusion occurred much more slowly. However, the actual timescale of viral fusion should be even shorter (seconds), due to the time delay of cytosolic diffusion and the temporal resolution of the microscope in our assay. Studies investigating the kinetics of viral fusion could benefit from new techniques with higher time resolution.

Previous studies identified multiple key elements of the VSV G protein, such as the fusion loop, membrane proximal region, and a few histidine residues. Despite increasing evidence from other fusion proteins implicating the essential role of the TM region in the fusion process, TM from VSV G was reported to be replaceable^18,22,37,38^. To clarify whether the TM region of VSV G is involved in the fusion process, we performed quantitative analysis using a cell-cell fusion assay. We found that the TM region of the VSV G protein was necessary for its role in the fusion process, like other fusion proteins. It has long been known that the TM region is not only an anchor on the membrane but also a mechanical element that exerts force on the lipid membrane. We noted that replacement of the VSV G TM significantly impaired its fusion activity, indicating that TMs from different fusogens are not equivalent. One possible explanation for this result is that the fusion function of TM relies on its cooperation with other domains/motifs. For example, a structural study showed that the helical bundle of SNARE proteins extends all the way to their TM regions, which suggests a cooperative working model of t-SNARE and v-SNARE TMs^39^. Moreover, mutation or replacement of SNARE TM regions impacts the stability or dilation of the initial fusion pore^19,20^. Viral fusion proteins function as a trimer, and more than one trimer works together during a fusion event^11^. Additionally, viral fusion proteins capture the host membrane via insertion of the fusion loop/peptide, which might also interact with TM region to rearrange lipid structure^40,41^. One possible explanation for defects of TM replacement is that other TM disrupts either the interactions among native VSV G TMs or the interaction between the TM and fusion loop. Thus, TMs from other fusion proteins cannot compensate for the functions of the native TM of VSV G. In this study, the most significant inhibitory effect was observed with the TM of the class I viral fusion protein HA, which holds the target membrane through the fusion peptide, a scenario that is very different from that of the bipartite fusion loops of VSV G^18,42^. The potential impact on the interaction between fusion loop-TM and fusion peptide-TM may result in the incompatibility of the HA TM in VSV G.

**Figure 7:**
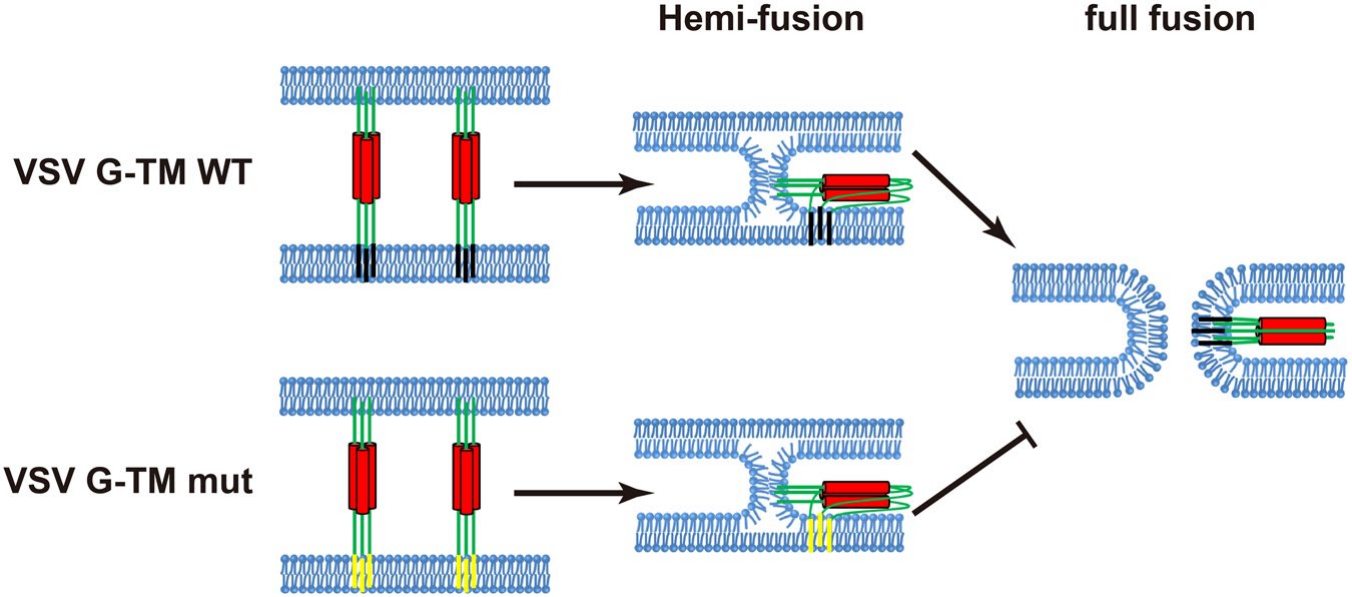
A model depicting the important role of TM in VSV G mediated fusion. VSV G pulls opposing membranes closer through conformational changes, and then two adjacent membranes undergo hemi-fusion; finally, the fusion pore opens, and two membranes merge into a single membrane (top panel). VSV G TM mutants initiate fusion, but the fusion process cannot proceed to full fusion; rather, the process is arrested at the hemi-fusion stage (bottom panel).

While exploring the fusion process in depth, we pinpointed TM function at the transition from hemi-fusion to full fusion. Cell-cell hemi-fusion assays captured the apparent hemi-fusion events caused by HA TM replacement, highlighting the blockage of the fusion process at the hemi-fusion stage. This finding provides new insight into the role of VSV G TM in membrane fusion. With TM replacement, the intact VSV G extracellular domain mediates the initiation step of fusion. However, once the process reaches the hemi-fusion stage, the incompatible TM cannot mediate the transition from hemi-fusion to full fusion (Fig. 7).

Mutation screening contingent on the homology between TMs from VSV G and HA allowed us to identify a few key residues in the VSV G TM region. The inadequate blocking effects of single mutations indicated the requirement of the entire TM sequence for VSV G fusion ability.

In summary, we developed a cell-cell fusion assay to analyze the fusion efficiency and timescale of VSV G induced cell-cell fusion. A cell-cell hemi-fusion assay implicated TM function in the hemi-fusion to full fusion transition. Lentivirus and recombinant VSV infection systems provided platforms to examine the physiological effects of TM function, which allowed us to establish a role for TM function in VSV G induced fusion, offering a new strategy for preventing VSV infection.

## Materials and Methods

### Cell culture

HEK293T (American type culture collection, ATCC) and HeLa (ATCC) cells were grown in DMEM medium (Gibco, USA, 12800017), CHO-K1 (ATCC) cells were grown in F12 medium supplemented with10% FBS (Gibco, USA, 16000-044) at 37 °C with 5% CO_2_.

### Western blotting

Protein samples extracted from cells were conducted SDS polyacrylamide gel electrophoresis and transferred onto Nitrocellulose membrane. Proteins on NC membrane were immunoblotted with anti-VSV G antibody (1:12,000; Santa Cruz, sc-66180), anti-p24 antibody (1:30,000; Sino Biological Inc.), or anti-β-actin antibody (1:10,000; Sigma-Aldrich, A5441) and then with secondary antibodies. Antibodies bound protein bands were detected by ECL plus western blot system (PerkinElmer, Waltham, MA, USA).

### Surface biotinylation assay

HeLa cells were transfected with wild-type or mutant VSV G expression plasmid pMD2.G (Addgene, 12259) as indicated for VSV G protein expression. 24 h after transfection, fresh DMEM medium was replaced and cells were incubated for another 2 h. Cells were washed twice with cold PBS buffer. Membrane proteins were labeled by 0.25 mM Sulfo-NHS-Biotin (Thermo scientific, #21217) in HBSS buffer at 4 °C for 15 min with gentle shaking. Cells were lysed with RIPA buffer followed by sonication. Biotinylated membrane proteins were pulled down by streptavidin agarose resin (Thermo fisher scientific, 20349) following the manufacturer’s instructions. Total and membrane localized VSV G proteins were detected by western blotting respectively and the relative ratio of membrane localized VSV G to total was determined by quantitative analysis using Image J program (NIH).

### Cell surface ELISA

2.5 × 10^4^/well HeLa cells were seeded into 98-well plate. After about 12 h, cells were transfected with VSV G born pMD2.G. At 24 h post transfection, fix cells by 4% formaldehyde for 10 min and then block cells by 3% BSA diluted in PBS for 30 min. Cells were incubated in 1:200 diluted VSV G antibody (Kerafast, E0010) at 37 °C for 2 h. After 3 times’ wash with PBS, cells were incubated in 1:1000 diluted HRP conjugated Goat anti-mouse antibody at 37 °C for 1 h. Rinse cells by PBS for 3 times and then add 200 ul TMB substrate solution (Beyotime Biotechnology). The absorbance at 650 nm was read by FlexStation 3 (Molecular Devices) at 5 min reaction.

### Cell-cell fusion assay

2 × 10^5^/well HeLa cells were seeded in 24-well plate. The next day, cells were co-transfected with 0.6 ug each of plasmid dsRed-nes and pMD2.G. On the same day, 7 × 10^4^/well HeLa cells which stably expressed ECFP-nls were seeded in 24-well plates with cover slips. 24 h after transfection, HeLa cells expressing VSV G and dsRed-nes were detached with citric saline solution (0.135 M KCl, 0.015 M sodium citrate). 7 × 10^4^/well of these cells were added to the wells that already containing HeLa cells with ECFP-nls expression for co-culture. After 16 h, cells were incubated in pH 6.0 MES or PBS buffer (pH 5.0) at room temperature for 1 min, then replaced with fresh DMEM medium and cultured for another 3 h at 37 °C. Cells were fixed with 4% formaldehyde for 10 min followed by three times’ wash with PBS and mounted with Fluoroshield (Sigma-Aldrich, F6182). Images were obtained on Olympus confocal microscope (FV1000). A fused cell is defined as one cell with red cytosol and multiple nuclei. Around 20 fields were randomly selected under microscope, fused cells and total cells with red cytosol were counted. The percentage of fused cells is calculated as the ratio of fused cells/syncytia to total cells with red cytosol.

### Live cell imaging

Cell fusion assay were performed as described above except cells were cultured in 35 mm glass bottom dishes. After 16 h co-culture, dishes were placed onto an open perfusion micro-incubator to keep the temperature at 37 °C. Cells were incubated in MES buffer (pH 6.0) for 1 min and then replaced with fresh DMEM medium. Images were continuously acquired every 15 s by Time Series configuration of the Zeiss confocal microscope (LSM780).

### Cell hemi-fusion assay

Cells stably expressing dsRed-nes were seeded onto 12 mm glass coverslips placed in 24-well plates. 6 × 10^5^/well CHO-K1 cells were seeded into 24-well plate and transfected with pMD2.G. 36 h after transfection, CHO-K1 cells were detached by citric saline solution and added onto cover slips with dsRed-nes expressing cells for co-culture. 16 hours later, cells were treated with pH 6.0 MES solution for 1 min and then incubated with normal growth medium. After 3 h incubation, cells were fixed with 4% formaldehyde for 10 min and then washed by PBS buffer for 3 times. Cover slips were incubated with 2 μg/ml FITC-Cholera Toxin β-subunit (Sigma-Aldrich, C1655) to label GM1 ganglioside. After three washes with PBS, the coverslips were mounted with Fluoroshield (Sigma-Aldrich, F6182). Confocal images were obtained on Zeiss confocal microscope (LSM780).

### Lentivirus preparation

1 × 10^6^/well HEK293 T cells were seeded into 12-well plate and transfected with 1.17 μg pLVTHM (Addgene 12247), 0.53 μg pMD 2.G (Addgene 12259) and 0.8 μg psPAX2 (Addgene 12260) plasmids. Next day, refresh the culture medium with Opti-MEM. After another 48 h, collect medium containing lentivirus particles.

### Lentivirus titration

1 × 10^5^/well HeLa cells were seeded into 24-well plate. Medium containing lentivirus with WT or mutant VSV G were diluted at 1:5, 1:25, 1:125, 1:625 respectively and added to HeLa cell. Refresh medium at 24 h after infection. After another 36 h, cells were fixed for imaging analysis by confocal microscope, or digested by trypsin and washed with PBS twice for analysis by flow cytometry.

### Recombinant VSV generation

Recombinant VSV were generated from pVSV and three helper plasmids (pBS-N, pBS-P, pBS-L) which encode VSV antigenomic RNA and VSV N/P/L proteins respectively as previously described^33^. The transcriptions of these four plasmids were mediated by T7 polymerase derived from pCAG-T7 plasmid. Briefly, all of these five plasmids were transfected into 293 T cells. Plasmid amounts were 2 μg pVSV, 0.25 μg pBS-N, 0.625 μg pBS-P, 0.25 μg pBS-L and 1.25 μg pCAG-T7. At 72 h post transfection, the culture DMEM medium was refreshed with Opti-MEM. After another 3–4 days culture, the plate was subjected to three round freeze-thawing (−80 °C, room temperature) and then cell lysate was centrifuged at 3000 rpm for 10 min. Viral supernatants were normalized by western-blot using VSV G antibody.

### Plaque assay

Viral stocks were 10-fold serially diluted in DMEM containing 2% FBS. For each dilution, 900 μl was added to Vero cells in 12-well plate. After 2 h incubation, medium containing virus was discarded and 1 ml of 1% low-melting agarose was added into each well, then the plate was incubated at 37 °C for 24 h. The infected cells were fixed with 4% formaldehyde for 1 h, followed by 1 h staining with 1% crystal violet. Visible plaques were counted and viral infection efficiencies were calculated.

### rVSV entry assay

Equal amount of WT and VSV G mutated rVSV were added into Vero cells. At 1 h post infection (p.i.), remove viral solution and wash cells with PBS twice. Cells seeded on cover slips were fixed by 4% formaldehyde for 10 min, and then permeabilized by 0.2% Triton X-100 for 10 min. After 30 min blocking by 3% BSA, cells were incubated in 1:200 diluted VSV G antibody (Santa Cruz, sc-66180) at 37 °C for 2 h followed by incubation in Alexa 488 conjugated secondary antibody at 37 °C for 1 h. After 1 ug/ml DAPI staining, coverslips were mounted for image capture.

At 1 h.p.i., cells were washed by PBS to remove surface docked virus. Total RNA was extracted from cell lysate by fast200 RNA extract kit (Fastagen). Real-time PCR were performed using One-step qPCR kit (TIANGEN) for viral RNA detection.

### Statistical analysis

The data was shown as mean ± SEM and statistical significant was determined by two tailed t-test. *P < 0.05, **P < 0.01, ***P < 0.001.

## Electronic supplementary material

Supplementary Information

Supplementary Video - vsv g mediated cell cell fusion
