# Supplementary material for: Vesicular stomatitis virus G protein transmembrane region is crucial for the hemi-fusion to full fusion transition
Source: Sci Rep. 2018 Jul 13;8:10669. doi: 10.1038/s41598-018-28868-y (PMC6045571; doi:10.1038/s41598-018-28868-y)
Supplement: Supplementary file 1 — Supplementary Information [file 41598_2018_28868_MOESM1_ESM.docx]

**Vesicular stomatitis virus G protein transmembrane region is crucial for the hemi-fusion to full fusion transition**

**Yali Ci ^1,2^, Yang Yang^1,2^, Caimin Xu^1,2^*, Lei Shi^1,2^***

1. State Key Laboratory of Medical Molecular Biology, Institute of Basic Medical Sciences, Chinese Academy of Medical Sciences & Peking Union Medical College, School of Basic Medicine, Beijing, China.

2. Department of Biochemistry and Molecular Biology, Institute of Basic Medical Sciences, Chinese Academy of Medical Sciences & Peking Union Medical College, School of Basic Medicine, Beijing, China.

*Caimin Xu: cmxu@ibms.pumc.edu.cn

*Lei Shi: shilei@ibms.pumc.edu.cn

Tel: +86 10 69156445

Short title: VSV G protein TM region is crucial for membrane fusion

**Supporting Information**

**Movie S1. Real-time imaging of cell-cell fusion mediated by VSV G protein.** The fusion happened soon after acidic buffer treatment, showing that the red fluorescent protein (dsRed-nes) diffused from one cell to adjacent cells with blue nuclei to form the fusion cell. Multiple rounds of cell fusion could be seen in the movie.


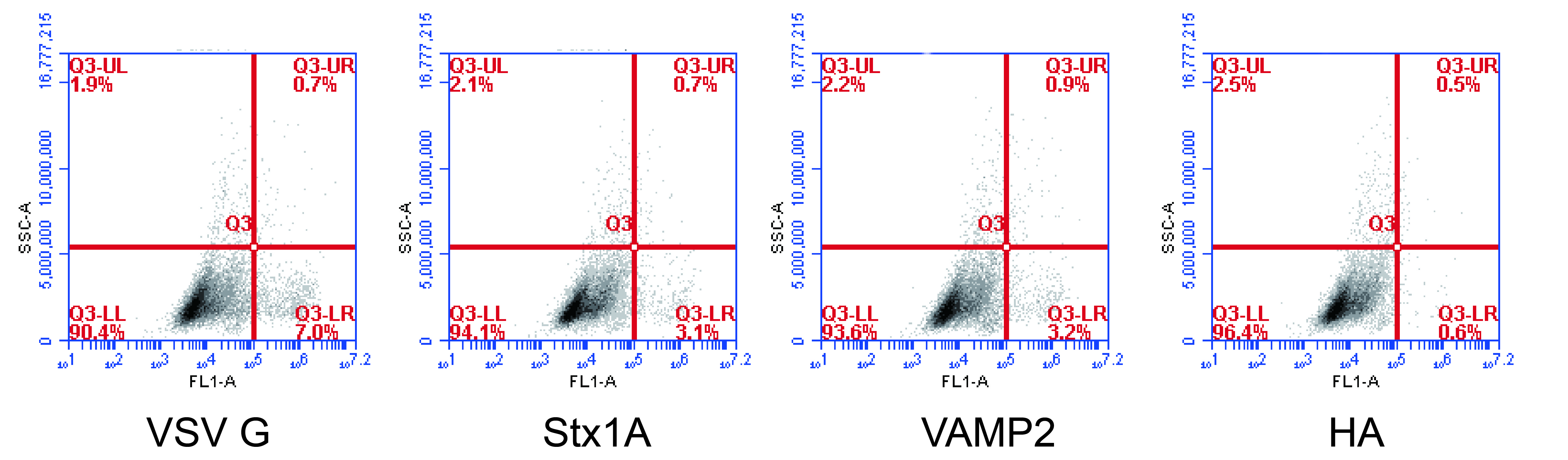


**Figure S2. TM replacements decreased infection efficiency of lentivirus.** HeLa cells infected by lentivirus with WT or chimeric VSV G were analyzed by cell flow cytometry. GFP is used as the reporter. GPF positive cells were lentivirus infected cells. The percentage of GFP positive cells were calculated as infection efficiency.


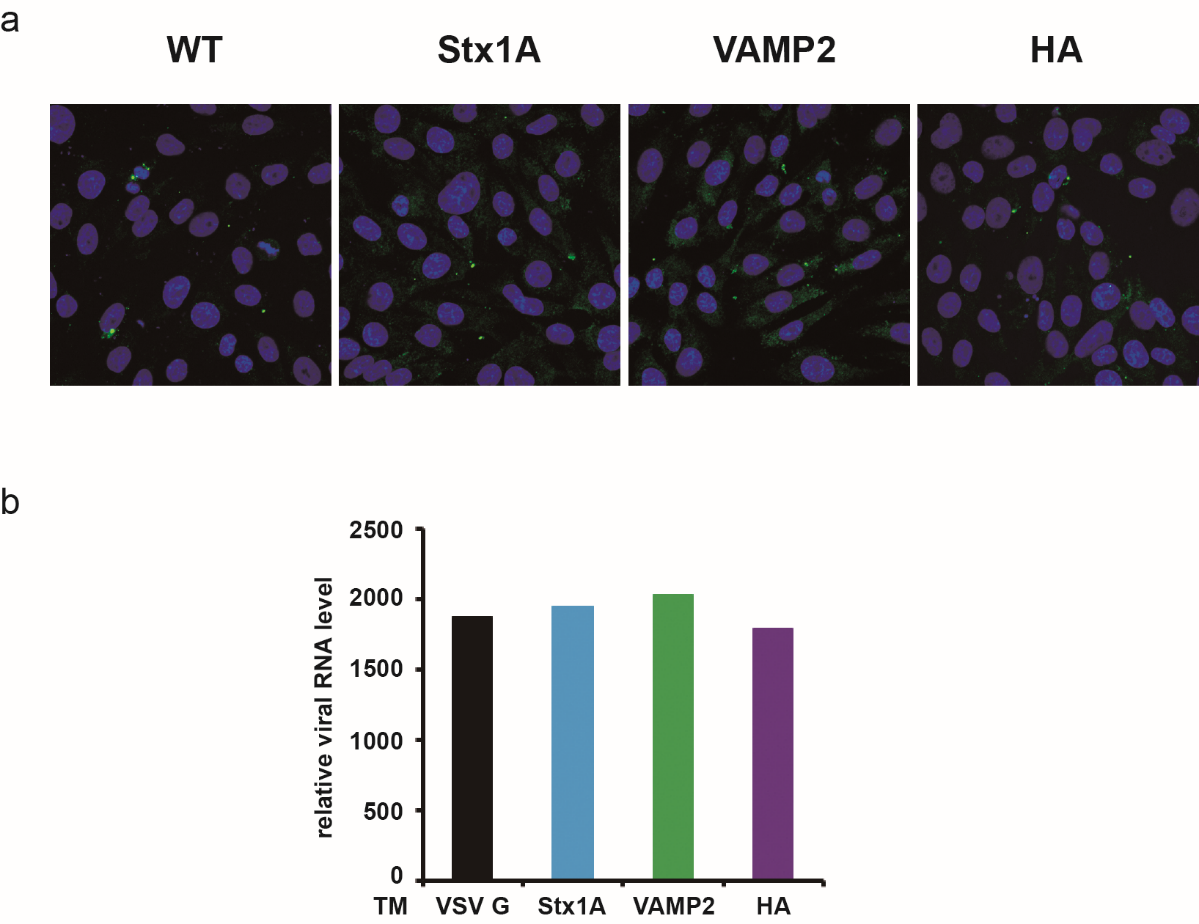


**Figure S3. TM replacement did not affect rVSV entry.** Vero cells were infected by rVSV carrying WT or mutated VSV G for 1h. (a) The infected cells were fixed and immunostained by VSV G antibody. (b) Viral RNA in cells was quantitated by real-time PCR.


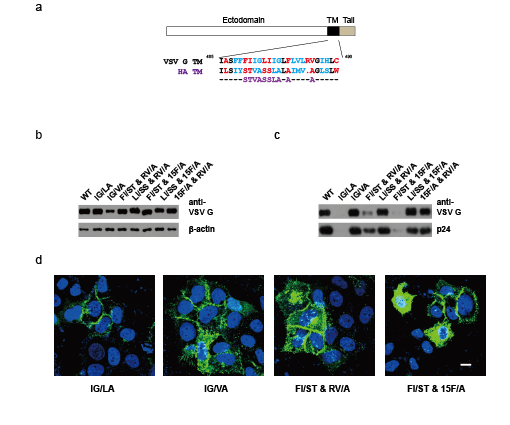


**Figure S4. Expression, virus package and localization of VSV G TM mutants.** (a) Amino acid sequence alignment of TM from VSV G and influenza HA. Black: identical, blue: homologous, red: non-conserved. (b) Detection of the expression of WT and mutated VSV G proteins in HeLa cell by western blot. IG/VA mutant displayed a significant lower expression level compared to WT VSV G. (c) Detection of the package of WT or mutated VSV G into lentivirus. Some mutants were packed onto lentivirus as well as WT. Three mutants (IG/LA, FI/ST & RV/A and FI/ST & 15F/A) had problem on lentivirus production. (d) The subcellular localization of the four VSV G mutants which had lower expression or defect in virus production. These mutants showed less expression or membrane localization in the cell. Scale bar, 10μm.


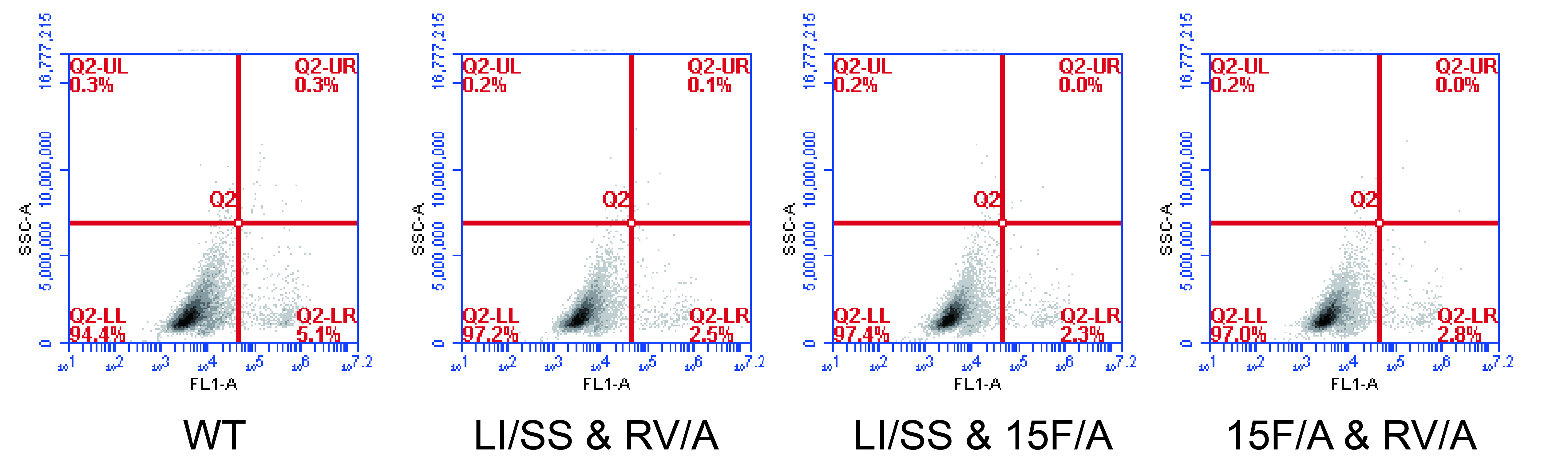


**Figure S5. Multi-site mutations in TM region attenuated infection efficiency of lentivirus.** HeLa cells infected by lentivirus with WT or mutated VSV G were analyzed by cell flow cytometry. Mutations in TM region decreased lentivirus infection efficiency.

**Table S1 Cell surface ELISA assay of VSV G (Mean±SE).** HeLa cells were transfected with pMD 2.G plasmids encoding WT or TM replaced VSV G and cell surface ELISA assay were performed to detect ectodomain of VSV G on plasma membranes. Similar absorbance at 650nm was obtained between cells expressing WT and mutated VSV G.

|  | control | WT | Stx1A | VAMP2 | HA |
| --- | --- | --- | --- | --- | --- |
| A650 | 0.14±0.01 | 0.53±0.03 | 0.55±0.03 | 0.63±0.06 | 0.61±0.05 |
